# Cost Burden, Readmission Dynamics, and Service Management in Psychiatric Care: A Financial Performance Analysis in a Romanian Public Hospital

**DOI:** 10.3390/healthcare14091204

**Published:** 2026-04-30

**Authors:** Laura Ioana Bondar, Roland Fazakas, Cris Virgiliu Precup, Denis Bogdan Butari, Florin Mihai Șandor, Ana-Liana Bouroș-Tataru, Elisaveta Ligia Piroș, Mariana Adelina Mariș, Liviu Gavrila-Ardelean, Florin Cornel Dumiter

**Affiliations:** 1 Doctoral School of Biomedical Sciences, University of Oradea, 410087 Oradea, Romania; bondar.lauraioana@student.uoradea.ro; 2 Department of Biology and Life Sciences, Faculty of Medicine, “Vasile Goldiș” Western University of Arad, 310025 Arad, Romania; precup.cris@uvvg.ro (C.V.P.); butari.denis-bogdan@uvvg.ro (D.B.B.); sandor.florin@uvvg.ro (F.M.Ș.); tataru.liana@uvvg.ro (A.-L.B.-T.); 3 Multidisciplinary Doctoral School, “Vasile Goldiș” Western University of Arad, 310025 Arad, Romania; 4 Department of General Medicine, Faculty of Medicine, “Vasile Goldiș” Western University of Arad, 310048 Arad, Romania; maris.mariana@uvvg.ro; 5 Prosthetic Dentistry, Faculty of Dental Medicine, “Vasile Goldiș” Western University of Arad, 310025 Arad, Romania; gavrila-ardelean.liviu@uvvg.ro; 6 Faculty of Economics, Informatics and Engineering, “Vasile Goldiș” Western University of Arad, 310025 Arad, Romania; dumiter.florin@uvvg.ro

**Keywords:** cost analysis, hospitalization, length of stay, mental disorders, psychiatry, readmission

## Abstract

**Highlights:**

**What are the main findings?**

- Acute and chronic psychiatric units differ substantially in care structure and cost profiles, with acute care showing higher daily costs but lower total cost per patient.
- Risk of 30-day readmission in acute psychiatry is driven by clinical severity (psychosis, multiple prior admissions) and a lack of coordinated discharge planning.

**What are the implications of the main findings?**

- Investment in transitional and community-based care may reduce preventable rehospitalizations and improve cost-efficiency in acute services.
- Optimizing long-stay resources and considering alternatives to prolonged institutionalization could mitigate the economic burden of chronic psychiatric hospitalization.

**Abstract:**

**Background/Objectives:** Psychiatric inpatient care varies substantially in its clinical goals, resource demands, and financial implications. Acute units focus on short-term crisis stabilization, whereas chronic units provide prolonged supervision for patients with persistent functional impairment. Limited evidence exists from Eastern Europe on how these differing service models impact both hospital costs and clinical outcomes such as early rehospitalization. This study aimed to compare the economic and operational performance of Acute versus Chronic Psychiatry and to identify predictors of 30-day readmission following acute psychiatric hospitalization. **Methods:** This retrospective observational study analyzed routinely collected data from a Romanian public hospital. All adult admissions to Acute and Chronic Psychiatry recorded between 1 January 2024 and 31 December 2024 were included. Standardized financial indicators were derived from administrative data, while clinical variables and readmission outcomes were extracted from electronic medical records. Between-group comparisons of economic and operational indicators were performed using *t*-tests. Multivariable logistic regression was used to determine independent predictors of 30-day readmission in Acute Psychiatry, reporting adjusted odds ratios (aOR) with 95% confidence intervals (CI). Model performance was evaluated with area under the curve (AUC), Hosmer–Lemeshow tests, and Nagelkerke R^2^. **Results:** Acute Psychiatry demonstrated significantly higher mean cost per bed-day (798.76 vs. 373.75 lei; *p* < 0.001), but a lower mean cost per patient due to shorter hospitalization (10.17 vs. 53.32 days). A total of 188 acute patients (13.7%) were readmitted within 30 days. No early readmissions occurred in Chronic Psychiatry, consistent with its long-stay care model. Independent predictors of readmission included psychotic disorder diagnosis (aOR = 1.62, 95% CI: 1.18–2.23), multiple prior admissions (aOR = 1.35, 95% CI: 1.18–1.54), shorter length of stay (LOS) (aOR = 0.88 per 5-day increase, *p* = 0.006), and absence of a post-discharge plan (aOR = 0.54, 95% CI: 0.39–0.76). Model discrimination was acceptable (AUC = 0.74). **Conclusions:** Acute and chronic psychiatric services differ markedly in cost structures and care pathways. Early rehospitalization is a clinically relevant outcome within acute psychiatric care and is influenced by both patient-level and continuity-of-care factors. Enhancing discharge coordination, expanding continuity-of-care strategies, and optimizing resource allocation toward community-based support may reduce early rehospitalizations while improving hospital cost-efficiency.

## 1. Introduction

Mental health disorders represent a major contributor to global disease burden, accounting for substantial morbidity, premature mortality, and socioeconomic loss [1,2]. Psychiatric hospitalization remains a critical component of care for individuals experiencing acute symptom exacerbation or chronic functional impairment [3,4,5]. However, worldwide reforms in mental health systems have increasingly emphasized cost-efficient hospital care combined with continuity of community-based services, aiming to reduce avoidable institutionalization and improve long-term recovery outcomes [6,7,8].

Significant variation exists between short-term acute psychiatric units and long-stay chronic wards in terms of treatment intensity, clinical outcomes, and resource consumption [9,10,11,12]. Acute hospitalization targets rapid stabilization of crises such as psychosis, suicidal behavior, or severe agitation, typically within brief admission windows. In contrast, chronic psychiatric care prioritizes the long-term supervision and structured support required for individuals with persistent disability [13,14,15]. In many Central and Eastern European settings, such long-stay “chronic” psychiatric units continue to play a substantial role in care delivery, often reflecting limited availability of community-based alternatives rather than a purely distinct therapeutic model. In contrast, in many Western European systems, inpatient stays of this magnitude (e.g., mean lengths exceeding 50 days) would be considered indicative of prolonged institutional care, highlighting ongoing challenges in the transition toward deinstitutionalized mental health services [4,16,17]. These divergent models produce distinct cost structures: acute units incur high daily operational expenses, whereas chronic care generates substantial cumulative institutional costs driven by extended length of stay (LOS) [18,19,20].

Early psychiatric rehospitalization—particularly within 30 days of discharge—is widely recognized as an indicator of clinical instability, insufficient treatment response, or breakdown in continuity of care [21,22,23]. Multiple predictive factors have been identified, including illness severity, psychotic disorders, frequent prior admissions, and premature discharge [24,25,26,27,28]. Equally important are organizational factors such as discharge planning, community support availability, and treatment adherence, including the use of long-acting injectable (LAI) antipsychotics [21,29,30,31]. Despite extensive international research, evidence from Eastern European psychiatric systems remains limited, particularly regarding integrated evaluations of both economic and clinical performance.

In Romania, public hospitals play a central role in psychiatric care delivery, yet persistent underfunding, low reimbursement tariffs, and limited community mental health infrastructure create challenges for both acute stabilization and long-term rehabilitation [32,33]. Understanding the economic and clinical implications of care pathways is therefore essential for evidence-based mental health policy.

This study addresses a critical gap by examining economic indicators and care pathway characteristics across Acute and Chronic Psychiatry within a Romanian public hospital, while identifying predictors of early psychiatric rehospitalization following acute discharge. By integrating financial analytics with multivariable risk modeling, the study aims to generate actionable evidence that can guide resource optimization and improve continuity of psychiatric care. The overarching goal is to support the development of a more efficient, patient-centered psychiatric service model aligned with ongoing European reforms.

## 2. Materials and Methods

### 2.1. Study Design and Setting

This retrospective observational study analyzed routinely collected clinical data from the Psychiatry Department of the Arad County Emergency Clinical Hospital, Romania. The department comprises two distinct inpatient units with different treatment profiles:

- Acute Psychiatry Unit—providing short-term crisis stabilization and intensive therapeutic interventions.
- Chronic Psychiatry Unit—providing long-term hospitalization for patients with persistent psychiatric symptoms requiring ongoing supervision and structured care.

The study analyzed all psychiatric admissions, discharges, and readmissions occurring between 1 January 2024 and 31 December 2024.

### 2.2. Study Population

All adult patients admitted to either the Acute or Chronic Psychiatry wards during the study period were eligible for inclusion. For analyses related to 30-day readmission, only patients discharged from the Acute Psychiatry ward were evaluated, as Chronic Psychiatry operates under a long-stay, non-episodic care model in which early readmission is not a directly comparable outcome.

#### 2.2.1. Inclusion Criteria

- Adults ≥18 years.
- Admission to Acute or Chronic Psychiatry between 1 January and 31 December 2024.
- Primary psychiatric diagnosis according to International Classification of Diseases, 10th Revision (ICD-10).
- Completion of an inpatient treatment episode within the study year.

#### 2.2.2. Exclusion Criteria

- Admissions for non-psychiatric primary medical conditions.
- Missing administrative, clinical, or cost data needed for analyses.
- Transfers to external psychiatric facilities (incomplete episodes).
- Planned readmissions (excluded from 30-day readmission analysis).

The unit of analysis in this study was the inpatient treatment episode. As such, individual patients could contribute more than one episode if they had multiple hospitalizations during the study period. Each admission was treated as a separate observation, reflecting real-world service utilization patterns. Because the primary outcome (30-day readmission) was defined at the level of discharge episodes, this approach was considered appropriate for the study objectives.

### 2.3. Data Collection and Variables

Data were retrieved retrospectively from the hospital’s electronic medical records and administrative databases. For each inpatient episode, standardized operational and financial indicators were extracted, including:

- Demographic characteristics: age, sex.
- Clinical variables: primary psychiatric diagnosis, LOS, number of previous admissions.
- Continuity-of-care indicators: documentation of a post-discharge plan, prescription of a LAI antipsychotic at discharge.
- Economic indicators: total cost per episode, mean cost per bed-day, mean cost per patient.
- Readmission outcome: unplanned readmission to Acute Psychiatry within 30 days of discharge, evaluated only for patients discharged from the Acute Psychiatry ward.

All data were fully anonymized prior to analysis, with patient identifiers removed by authorized hospital personnel.

### 2.4. Statistical Analysis

All statistical analyses were performed using IBM SPSS Statistics, version 29 (IBM Corp., Armonk, NY, USA).

Continuous variables were summarized as mean ± standard deviation (SD) or median (interquartile range, IQR), as appropriate based on distribution, and compared using independent-samples *t*-tests or Mann–Whitney U tests accordingly. The distribution of continuous variables was assessed for normality using the Shapiro–Wilk test, supplemented by visual inspection of Q–Q plots. Homogeneity of variance was evaluated using Levene’s test. When assumptions of equal variances were not met, Welch’s *t*-test was applied. Parametric tests were applied when these assumptions were satisfied; otherwise, appropriate non-parametric alternatives were used. Categorical variables were presented as frequencies and percentages, and analyzed using chi-square (χ^2^) tests with effect sizes reported as Cramer’s V where applicable.

Because Acute and Chronic Psychiatry represent structurally different models of inpatient care, inferential comparison of 30-day readmission rates between wards was not considered appropriate. Accordingly, readmission analyses were conducted exclusively within the Acute Psychiatry cohort, where discharge and subsequent readmission represent a clinically meaningful episodic outcome.

Univariable analyses were initially performed to identify factors associated with 30-day readmission in Acute Psychiatry. Variables with *p* < 0.10 in univariable analyses, together with variables considered clinically relevant based on prior evidence and domain knowledge, were entered into a multivariable logistic regression model to determine independent predictors of early rehospitalization. This combined approach was used to reduce the risk of excluding potentially important confounders that may not reach statistical significance in univariable testing. Adjusted odds ratios (aOR) with 95% confidence intervals (CI) were reported. Continuous predictors were rescaled to improve interpretability of effect estimates. Specifically, LOS was modeled per 5-day increase, and age was modeled per 10-year increase.

Prior to model estimation, key assumptions of logistic regression were assessed. The relationship between continuous predictors and the logit of the outcome was examined to ensure approximate linearity. Multicollinearity was evaluated using variance inflation factors (VIF), with all values below commonly accepted thresholds (VIF < 5), indicating no evidence of problematic collinearity. Sensitivity analyses using alternative model specifications were performed and yielded consistent results, supporting the robustness of the findings.

Model performance was evaluated using the Hosmer–Lemeshow goodness-of-fit test (calibration), Nagelkerke R^2^ (explanatory power), and the receiver operating characteristic (ROC) curve with area under the curve (AUC; discrimination).

All statistical tests were two-tailed, and a *p*-value < 0.05 was considered statistically significant.

### 2.5. Cost Analysis Methodology

Cost evaluation was conducted from the hospital provider perspective using retrospective administrative financial data. Total ward-level costs included both direct and indirect components. Direct costs comprised personnel expenses (salaries and social contributions), medications and medical supplies, and diagnostic and therapeutic procedures. Indirect and shared costs—including food, utilities, administrative services, and overhead infrastructure—were allocated at the ward level proportionally based on total bed-days, in accordance with standard hospital cost-accounting practices.

The following standardized economic indicators were calculated:

- Mean cost per bed-day: total annual cost of each ward divided by the total number of occupied bed-days.
- Mean cost per patient: total annual ward-level costs divided by the number of unique patients treated within the study period.

The mean cost per patient represents an aggregate annual indicator and does not correspond to the cost of a single hospitalization episode. Therefore, it should be interpreted as a population-level metric and considered alongside LOS and cost per bed-day when comparing care models.

Tariffs reimbursed by the National Health Insurance House (CNAS) were obtained based on the contracted daily rate for each ward. The cost–tariff gap was computed as:

Cost–Tariff Gap = Mean Cost per Bed-Day − CNAS Tariff per Bed-Day

A positive value indicates that the cost of care exceeds reimbursement (potential underfunding), whereas a negative value indicates that reimbursement exceeds the daily cost of care.

All values are expressed in Romanian lei (RON). Cost data were complete for all included episodes; therefore, no imputation procedures were required.

### 2.6. Ethical Considerations

The study was conducted in accordance with the ethical principles outlined in the Declaration of Helsinki and relevant institutional and national data protection regulations, including the EU General Data Protection Regulation (GDPR).

This study was designed as a retrospective analysis of routinely collected clinical and administrative data from the year 2024, which were originally recorded as part of standard patient care and hospital operations. Data were obtained from hospital information systems as well as archived paper-based medical records. Ethical approval for the secondary use of these anonymized data for research purposes was obtained from the Ethics Committee of Arad County Emergency Clinical Hospital (Approval No. 116/10 February 2026).

No data extraction or analysis for research purposes was performed prior to obtaining ethical approval. The study involved no direct patient contact, no medical interventions, and no collection of identifiable personal information, as it is based exclusively on retrospectively extracted anonymized hospital records.

All data were securely stored and accessible only to the research team to ensure full confidentiality and compliance with data protection standards.

### 2.7. Hypotheses of the Study

Based on previous evidence highlighting variability in psychiatric care pathways, healthcare resource utilization, and factors contributing to early psychiatric rehospitalization, the present study examined the following hypotheses:

1. Acute and Chronic Psychiatry differ significantly in economic and operational performance, with Acute Psychiatry demonstrating higher cost per bed-day but shorter LOS, and Chronic Psychiatry demonstrating prolonged hospitalization resulting in a higher total cost per patient.
2. Early psychiatric readmissions predominantly occur following discharge from Acute Psychiatry, reflecting a higher level of clinical instability and challenges in post-discharge continuity of care.
3. Clinical characteristics, particularly psychotic disorder diagnosis and history of multiple prior admissions, increase the likelihood of 30-day readmission among patients discharged from Acute Psychiatry.
4. Organizational care factors, including LOS and documentation of a post-discharge plan, are significant predictors of early readmission, with shorter hospitalization and absence of coordinated follow-up increasing the risk.
5. Treatment with LAI antipsychotics at discharge is associated with reduced odds of 30-day readmission, although the strength of this relationship may vary based on patient clinical profiles.

## 3. Results

This section presents the financial performance, readmission patterns, and predictors of early rehospitalization across acute and chronic psychiatric services.

### 3.1. Financial Performance in Acute Psychiatric Care

In 2024, the Acute Psychiatry unit admitted 1372 patients, generating a total of 13,939 bed-days. The overall expenditure for this unit was 11,134,185.68 lei, corresponding to a mean cost of 798.76 lei per bed-day. As shown in Table 1, personnel expenses accounted for 66.5% of total spending, emphasizing the labor-intensive profile of acute mental health care. The mean cost per patient was 8126.95 lei, and the average LOS was 10.17 days, reflecting a high patient turnover consistent with crisis stabilization and short-term treatment objectives.

### 3.2. Financial Performance in Chronic Psychiatric Care

In 2024, the Chronic Psychiatry ward treated 81 patients, with a cumulative 4319 bed-days and total expenditure of 1,614,227.42 lei. The mean cost per bed-day was 373.75 lei, which is substantially lower than in acute care and reflects reduced intervention intensity. However, as presented in Table 2, the mean cost per patient reached 19,928.73 lei, driven by a prolonged mean LOS of 53.32 days. Personnel-related expenses represented 62.4% of the total cost, confirming that labor remains the primary cost driver even in long-term psychiatric hospitalization.

### 3.3. Comparison of Key Economic Indicators

A comparative analysis between the two psychiatric service types revealed substantial differences in financial and operational performance (Table 3). Acute Psychiatry reported higher daily costs (798.76 lei vs. 373.75 lei) and a significantly shorter LOS (10.17 vs. 53.32 days), reflecting the distinct clinical profile of short-term crisis interventions. In contrast, Chronic Psychiatry generated a much higher total cost per patient (19,928.73 lei vs. 8126.95 lei), primarily driven by prolonged hospitalization.

The cost–tariff difference represents the relationship between actual hospital costs and reimbursement levels, where positive values indicate that costs exceed reimbursement and negative values indicate that reimbursement exceeds the daily cost of care.

After comparing the two units, independent-samples *t*-tests demonstrated that all differences in economic and operational indicators were statistically significant and of very large magnitude (Table 4). Acute Psychiatry showed a significantly higher cost per bed-day, whereas Chronic Psychiatry incurred a greater cost per patient, explained by the extensive LOS. Additionally, LOS differed markedly between services, confirming that the two units operate under fundamentally distinct clinical and resource utilization models.

**Figure 1:**
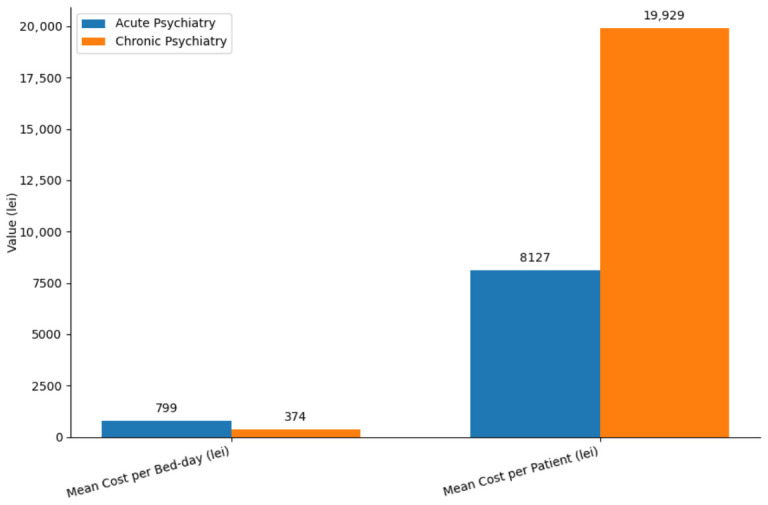
Cost comparison between acute and chronic psychiatry: mean cost per bed-day and mean cost per patient. The cost–tariff gap represents the difference between mean cost per bed-day and the reimbursement tariff; positive values indicate that costs exceed reimbursement (potential underfunding), whereas negative values indicate that reimbursement exceeds the cost of care.

These differences are visually illustrated in Figure 1, which highlights the contrasting economic profiles of the two service types.

**Figure 2:**
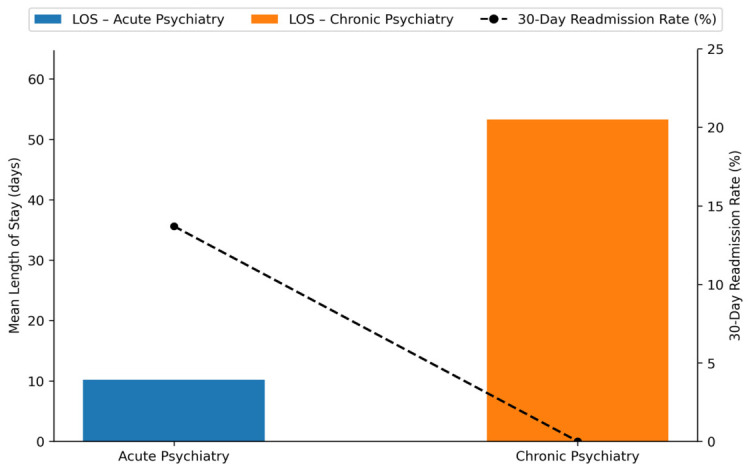
Clinical performance indicators for Acute vs. Chronic Psychiatry. Mean length of stay (LOS) is shown as bars (blue = Acute Psychiatry; orange = Chronic Psychiatry), while the dashed line represents the 30-day readmission rate (%).

Figure 2 further demonstrates the clinical divergence, showing shorter LOS in acute care but higher early readmission rates.

### 3.4. Readmission Analysis

During the study period, 188 patients from Acute Psychiatry were readmitted within 30 days of discharge, corresponding to a 30-day readmission rate of 13.70%. In contrast, no early rehospitalizations were recorded in the Chronic Psychiatry ward, consistent with its long-stay treatment model. Descriptive results are presented in Table 5. Given the structural differences between care models and the absence of readmission events in Chronic Psychiatry, inferential statistical comparison (including Fisher’s Exact Test) was not considered appropriate.

These findings should be interpreted in the context of the different care models represented by the two wards. Acute Psychiatry operates as an episodic, short-stay service with discharge and potential readmission as part of the clinical pathway, whereas Chronic Psychiatry provides long-term inpatient care with prolonged hospitalization.

Accordingly, the absence of 30-day readmissions in Chronic Psychiatry reflects the structural characteristics of the care model rather than a directly comparable outcome. Therefore, readmission rates are presented descriptively, and no inferential statistical comparison between wards was performed.

Subsequent analyses of factors associated with 30-day readmission were conducted exclusively within the Acute Psychiatry cohort, where this outcome is clinically meaningful.

### 3.5. Univariable Factors Associated with 30-Day Readmission

Univariable analyses were conducted to compare clinical and service-related factors between readmitted and non-readmitted patients in the Acute Psychiatry ward, with results presented using both descriptive statistics and inferential testing (Table 6). Patients with 30-day readmission had a higher mean number of previous hospitalizations, a greater proportion of psychotic disorder diagnosis, shorter index LOS, and less likely to have a documented post-discharge plan, highlighting the combined influence of clinical severity and continuity-of-care gaps on early relapse risk.

### 3.6. Multivariable Logistic Regression Model

All variables with *p* < 0.10 in univariable tests, together with clinically relevant co-variates, were entered into a multivariable logistic regression model to identify independent predictors of 30-day readmission in Acute Psychiatry. The final model included: age, sex, number of previous admissions, primary diagnosis (psychotic vs. other), LOS, documentation of a post-discharge plan, and prescription of a LAI antipsychotic at discharge.

As shown in Table 7, patients with a greater number of previous admissions had significantly higher odds of 30-day readmission (aOR = 1.35, 95% CI: 1.18–1.54, *p* < 0.001), and those diagnosed with a psychotic disorder were also at increased risk (aOR = 1.62, 95% CI: 1.18–2.23, *p* = 0.003). A longer index LOS demonstrated a protective effect, with each additional 5 days reducing the likelihood of readmission (aOR = 0.88, 95% CI: 0.80–0.96, *p* = 0.006). Likewise, documentation of a post-discharge plan significantly lowered readmission risk (aOR = 0.54, 95% CI: 0.39–0.76, *p* < 0.001).

In contrast, LAI antipsychotic treatment at discharge suggested a reduction in readmission risk, although this association did not reach statistical significance (aOR = 0.79, 95% CI: 0.58–1.08, *p* = 0.140). Age and sex were not significant predictors. Overall, the model demonstrated acceptable calibration and discrimination (Table 8), supporting the relevance of these clinical and organizational factors in relation to early readmission risk in acute psychiatric care. No evidence of multicollinearity was identified among predictors, with all VIF values below 2.

Given the relatively low prevalence of 30-day readmission, threshold-based performance metrics were considered alongside AUC. The model demonstrated higher specificity than sensitivity, indicating better identification of non-readmitted than readmitted patients.

**Figure 3:**
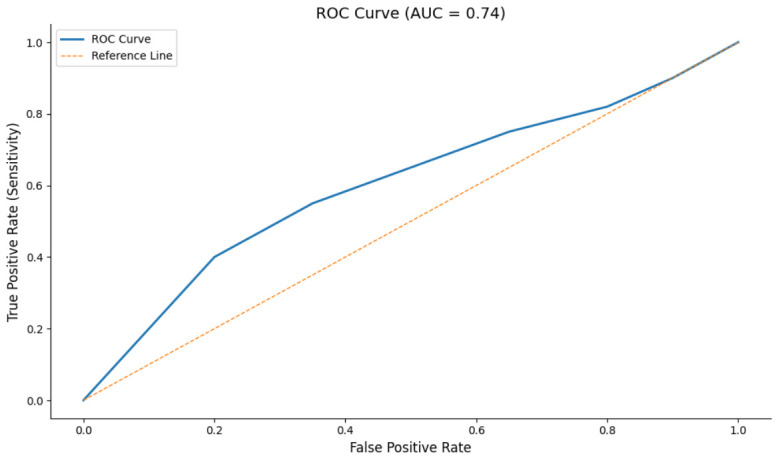
ROC curve for the logistic regression model predicting 30-day readmission in Acute Psychiatry (AUC = 0.74). The diagonal line represents chance-level discrimination.

Model discrimination was further examined using the receiver operating characteristic (ROC) curve, which demonstrated acceptable discrimination (AUC = 0.74), as shown in Figure 3.

Overall, the findings indicate clear differences in cost structures and resource utilization between acute and chronic psychiatric services, reflecting their distinct care models. The acute unit demonstrated higher efficiency but was characterized by measurable early rehospitalizations following discharge, while the chronic unit showed prolonged LOS and higher cumulative cost per patient. The multivariable model further highlighted that early readmission risk in acute psychiatry is mainly associated with clinical chronicity and gaps in continuity of care.

## 4. Discussion

This study provides a comprehensive evaluation of financial performance and clinical outcomes across two distinct psychiatric care pathways in a Romanian public hospital. The findings were consistent with the study hypotheses, suggesting meaningful differences between acute and chronic psychiatric services in terms of economic burden, resource utilization, and early readmission dynamics.

### 4.1. Economic Divergence Between Acute and Chronic Care Models

The present study suggests that Acute Psychiatry was characterized by substantially higher daily treatment costs, reflecting the intensive resource demands of crisis stabilization and short-term interventions. However, due to markedly shorter hospitalization durations, the overall cost per patient in the acute unit remained significantly lower than in Chronic Psychiatry. In contrast, prolonged admissions in the chronic ward generated a much higher cumulative financial burden despite lower mean costs per bed-day.

These findings are in line with previous international reports indicating that long-stay psychiatric units consume a disproportionate share of hospital resources, largely driven by fixed staffing requirements and ongoing social-care needs rather than high-intensity clinical procedures [18,34]. The observed positive cost–tariff gap in acute care indicates that the actual cost of care exceeds reimbursement, suggesting potential structural underfunding of high-intensity psychiatric services. Conversely, the negative cost–tariff gap in Chronic Psychiatry reflects lower daily costs relative to reimbursement, likely due to reduced intervention intensity despite prolonged hospitalization [35,36,37].

### 4.2. Early Readmission as a Marker of Clinical Instability

Early readmissions occurred exclusively in the acute care setting, with a 30-day rehospitalization rate of 13.7%. This value is consistent with European reports indicating that 10–20% of patients discharged from acute psychiatric units experience rapid relapse and return to care [38,39,40]. These findings support the clinical reality that acute hospitalization frequently represents a short-term interruption within a fluctuating chronic disease course, rather than a complete resolution of symptomatology [24,36].

However, this observation should not be interpreted as a direct comparison of clinical performance between Acute and Chronic Psychiatry. The two units represent fundamentally different models of care, with Acute Psychiatry functioning as an episodic, short-stay service, whereas Chronic Psychiatry operates as a long-stay, non-episodic care pathway.

The absence of early rehospitalizations in the Chronic Psychiatry ward reflects the structural characteristics of long-term inpatient care, where prolonged hospitalization reduces exposure to early post-discharge risk. This should not be interpreted as evidence of superior clinical outcomes, but rather as a consequence of different discharge dynamics and care pathways [21,22,24,39,41]. Accordingly, 30-day readmission is best understood as an outcome specific to acute psychiatric care and not as a directly comparable performance indicator across fundamentally different service models [18,42,43,44].

Taken together, these results reinforce the importance of robust transitional care pathways, including early community follow-up and psychosocial support interventions, to mitigate the heightened vulnerability period immediately following discharge from acute psychiatric hospitalization [40,45,46].

### 4.3. Drivers of Early Psychiatric Rehospitalization

Multivariable analysis indicates that both clinical and organizational characteristics significantly influenced early psychiatric rehospitalization outcomes. A higher number of previous admissions and the presence of a psychotic disorder diagnosis were independently associated with increased 30-day readmission risk. These findings are consistent with prior studies identifying recurrent hospitalization and psychosis as markers of high clinical complexity, vulnerability to rapid decompensation, and persistent functional impairment [47,48,49].

A shorter index LOS was also associated with greater likelihood of readmission, which may reflect insufficient clinical stabilization at discharge. Similar associations have been reported across European health systems, highlighting the tension between reducing LOS for efficiency and ensuring clinical readiness for discharge [50,51,52,53,54].

Importantly, one of the strongest protective factors identified in this study was the presence of a documented post-discharge plan. The absence of coordinated transition-of-care measures significantly increased the risk of rapid relapse, reinforcing evidence that early community engagement and structured follow-up are crucial components of continuity-of-care strategies [55,56,57,58,59]. This is particularly relevant in regions where outpatient mental health services and psychosocial supports remain unevenly distributed.

Although the prescription of LAI antipsychotics demonstrated a trend toward lower readmission risk, the association did not reach statistical significance. This finding should be interpreted in the context of potential confounding by indication, as LAIs are often preferentially prescribed to patients with more severe illness or a history of poor treatment adherence [60,61,62,63,64,65].

Overall, the results support a multidimensional conceptualization of readmission risk, where symptom severity, chronicity, and system-level care coordination jointly influence the likelihood of rapid psychiatric relapse.

Although the model demonstrated acceptable discrimination, its lower sensitivity compared to specificity suggests that it is more effective in identifying patients at low risk of readmission than in accurately predicting all high-risk cases. This limitation highlights the complexity of psychiatric relapse and the need for incorporating additional clinical and psychosocial factors in future predictive models.

### 4.4. Protective Trends Associated with LAI Antipsychotics

Although LAI antipsychotics did not reach statistical significance as an independent predictor of reduced 30-day readmission risk in the fully adjusted model (*p* = 0.140), the association showed a clinically relevant protective trend. This finding aligns with extensive evidence demonstrating that LAIs can improve medication adherence, reduce relapse frequency, and enhance long-term outcomes in schizophrenia-spectrum disorders [66,67,68].

The absence of significance may be attributed to limited statistical power, variability in clinical indications for LAI initiation, or potential confounding related to illness severity. Future research using larger samples or subgroup analyses focused on high-risk diagnostic categories may better elucidate the magnitude of benefit.

Nevertheless, the results highlight an actionable target for service improvement: expanding access to LAIs and integrating them into individualized relapse-prevention strategies—particularly in settings where adherence challenges and fragmented continuity of care contribute substantially to early rehospitalization risk.

### 4.5. Implications for Policy and Resource Allocation

The findings have notable implications for strategic mental health planning and financing. The substantial cumulative costs observed in long-stay psychiatric hospitalization suggest a need for targeted resource redistribution toward community-based residential and social-support services, which have demonstrated cost-effectiveness in managing chronic and functionally impaired patients outside the hospital environment.

Conversely, the elevated risk of early relapse following acute psychiatric discharge underscores the necessity of investment in transitional and continuity-of-care programs, such as structured case management, early outpatient follow-up, crisis intervention teams, and family or caregiver psychoeducation. Strengthening these pathways could reduce avoidable hospital returns and improve long-term recovery trajectories.

Furthermore, the positive cost–tariff gap in acute care indicates a misalignment between reimbursement models and actual resource utilization, potentially disincentivizing high-intensity services that are essential for crisis stabilization. Integrating performance-linked or needs-adjusted financing mechanisms may enhance sustainability while promoting efficient clinical outcomes.

Overall, these policy implications are consistent with contemporary European mental health system reforms that prioritize deinstitutionalization, cost-efficient resource allocation, and robust community integration as core pillars for optimized psychiatric care delivery.

### 4.6. Strengths and Limitations

This study has multiple methodological strengths. First, it utilized a complete inpatient cohort from a full operational year, ensuring high internal validity and eliminating sample selection bias. This approach provides a realistic representation of psychiatric care utilization and economic burden in routine clinical practice. Second, the simultaneous evaluation of service performance, cost efficiency, and readmission outcomes offers an integrated view of how clinical and financial pressures interact within different psychiatric care models. This dual analytical approach remains underrepresented in Romanian and Eastern European mental health research. Third, the statistical framework included univariable and multivariable modeling, with performance indicators demonstrating acceptable predictive discrimination (AUC = 0.74) and good calibration, adding confidence to the identified determinants of early relapse.

Despite these strengths, several limitations must be acknowledged. A key methodological limitation is that Acute and Chronic Psychiatry represent non-equivalent models of care, with fundamentally different admission patterns, LOS, and discharge dynamics. As a result, 30-day readmission cannot be interpreted as a directly comparable performance indicator between wards, and differences in readmission rates primarily reflect structural characteristics of the care models rather than equivalent patient outcomes.

The single-center design restricts the generalizability of findings to other regions with different psychiatric care structures, funding policies, or community support availability. The study was conducted in a large public county emergency hospital that functions as a regional referral center and broadly reflects the organization of psychiatric inpatient care in Romania, which remains predominantly hospital-based with limited community mental health infrastructure. However, regional differences in resource availability, patient case-mix, and service organization may influence the extent to which these findings are generalizable to other settings. Although the study included comprehensive hospital-based variables, it lacked information on key psychosocial determinants known to drive readmission risk, such as housing insecurity, social support networks, substance use disorders, treatment adherence history, and socioeconomic status. In addition, the “post-discharge plan” variable reflects documentation of intended follow-up rather than confirmed continuity of care, as no data were available on whether patients attended scheduled outpatient appointments or received subsequent community-based services after discharge. These unmeasured factors could partially explain residual variance in the regression model.

Furthermore, clinical characterization relied on administrative diagnoses, without access to standardized symptom severity measures or functional status indicators, which limits precision in quantifying illness acuity at discharge. The analysis also focused on 30-day readmission events, which capture short-term instability but do not reflect long-term relapse patterns or avoidable emergency care episodes managed outside hospitalization. Additionally, planned readmissions were excluded, which may reduce comparability with systems where such admissions are included in performance benchmarking.

Finally, while cost data were fully available and accurate, they reflect only the hospital provider perspective, excluding broader health-economic implications such as outpatient resource consumption, caregiver burden, or indirect societal costs. Additionally, the use of mean cost per patient as an aggregate annual indicator does not correspond to the cost of individual hospitalization episodes, which may limit direct comparability with studies using episode-based costing approaches.

Although key assumptions of the logistic regression model were assessed, including linearity of continuous predictors and absence of multicollinearity, the observational nature of the data and potential model specification choices may still introduce residual uncertainty in the estimated associations.

### 4.7. Future Directions

Building on the present findings, several directions for future research and psychiatric service development are warranted. First, the implementation and formal evaluation of structured discharge planning protocols could play a critical role in reducing early rehospitalizations. Approaches incorporating immediate community follow-up appointments, crisis support availability, and family engagement strategies may help ensure clinical stability during the transition from inpatient to outpatient care. Future research should also incorporate more granular measures of continuity of care, including verification of post-discharge follow-up attendance, timing of outpatient contact, and engagement with community-based services, in order to distinguish between planned and effectively delivered transitional care.

Another priority is the expansion of LAI antipsychotic therapy among eligible patients. Although the current study observed a non-significant protective trend, prior research suggests strong potential benefits in improving medication adherence and preventing relapse. Increasing clinician awareness, patient education, and access to LAIs may further clarify and enhance their role in real-world clinical settings.

Strengthening community-based aftercare services also emerges as a key opportunity. Multidisciplinary follow-up models—including assertive outreach, case management, home-based support, and telepsychiatry—may mitigate the heightened vulnerability period immediately following discharge. Evaluating these interventions in routine practice may identify scalable strategies to reduce preventable readmissions.

Future work should also incorporate a more comprehensive assessment of social determinants of mental health, such as housing stability, employment, and substance use. Integrating these contextual factors into predictive models may improve identification of high-risk patients and facilitate more individualized care planning. In addition, health-economic modeling could assess the cost–benefit implications of reducing prolonged institutionalization while reallocating resources toward community rehabilitation.

Finally, expanding analyses to multi-center and longitudinal cohorts would improve generalizability and allow examination of long-term outcomes beyond the initial 30-day window. Advanced analytical approaches, including machine-learning-based risk prediction, may further refine early detection of relapse risk and support data-driven decision-making.

Collectively, these future research directions highlight the need for integrated, patient-centered psychiatric care systems that balance hospitalization requirements with sustained community support, ultimately improving outcomes while optimizing the use of healthcare resources.

## 5. Conclusions

This study provides a comprehensive assessment of clinical and economic performance in two distinct psychiatric care models within a large public hospital. Acute Psychiatry demonstrated high operational efficiency with intense resource utilization over short inpatient stays but faced a notable burden of early rehospitalizations, reflecting inherent clinical instability and challenges in transitional care. In contrast, Chronic Psychiatry incurred substantially higher cumulative costs per patient due to prolonged hospitalization, reflecting a fundamentally different long-stay care pathway in which short-term readmission is not directly comparable to acute care settings.

The multivariable analysis identified key predictors of 30-day readmission, including clinical chronicity factors such as psychotic disorder diagnosis and repeated prior hospitalizations, as well as organizational care characteristics such as shorter LOS and the absence of a structured post-discharge plan. These results highlight the potential importance of both patient complexity and system-level continuity of care in shaping outcomes following acute psychiatric treatment.

Taken together, the findings suggest that strengthening discharge coordination, enhanced outpatient follow-up, and expanded access to adherence-supportive treatments such as LAI antipsychotics may help reduce early rehospitalizations. Additionally, optimizing resource allocation through the gradual reduction in long-stay institutionalization and the strengthening of community-based mental health services may improve both cost-efficiency and patient outcomes.

By integrating economic and clinical perspectives, this study underscores the importance of data-driven service planning to achieve a resilient and patient-centered psychiatric care system. Continued research incorporating broader determinants of mental health and multicenter validation will be essential to guide policy and ensure sustainable improvements across the continuum of psychiatric care.

## Figures and Tables

**Table 1:** Key financial indicators for Acute Psychiatry.

Indicator	Value
Number of patients	1372
Bed-days	13,939
Total cost	11,134,185.68 lei
Mean cost per bed-day	798.76 lei
Personnel cost share	66.5%
Mean cost per patient	8126.95 lei
Mean LOS	10.17 days

**Table 2:** Key financial indicators for Chronic Psychiatry.

Indicator	Value
Number of patients	81
Bed-days	4319
Total cost	1,614,227.42 lei
Mean cost per bed-day	373.75 lei
Personnel cost share	62.4%
Mean cost per patient	19,928.73 lei
Mean LOS	53.32 days

**Table 3:** Comparative financial performance indicators for Acute vs. Chronic Psychiatry.

Indicator	Acute Psychiatry	Chronic Psychiatry
Bed-days	13,939	4319
Mean cost per bed-day	798.76 lei	373.75 lei
Tariff per bed-day (CNAS)	593 lei	592 lei
Cost–Tariff	+205.76 lei	−218.25 lei
Mean cost per patient	8126.95 lei	19,928.73 lei
Mean LOS	10.17 days	53.32 days

**Table 4:** Independent-samples *t*-test results comparing Acute vs. Chronic Psychiatry.

Variable	Mean Difference	t-Value	df	*p*-Value	Effect Size (Cohen’s d)
Mean cost per bed-day	+425.01 lei	14.52	1451	<0.001	1.20
Mean cost per patient	−11,801.78 lei	12.37	1451	<0.001	1.03
Mean LOS	−43.15 days	31.48	1451	<0.001	2.63

**Table 5:** Readmission outcomes within 30 days by psychiatric ward type.

Outcome	Acute Psychiatry	Chronic Psychiatry
Total patients	1372	81
30-day readmissions	188	0
30-day readmission rate	13.70%	0.00%

**Table 6:** Univariable comparison of predictors of 30-day readmission in acute psychiatry.

Predictor	Readmitted Patients	Non-Readmitted Patients	Statistical Test	*p*-Value
Number of previous admissions	3.4 ± 2.1	2.1 ± 1.6	*t*-test	*p* = 0.003
Primary diagnosis: psychotic disorder	112 (59.6%)	512 (43.2%)	χ^2^ test	*p* = 0.028
LOS (days)	8 (IQR 6–12)	10 (IQR 7–14)	Mann–Whitney U	*p* = 0.014
Post-discharge plan documented	78 (41.5%)	742 (62.7%)	χ^2^ test	*p* < 0.001
LAI prescribed at discharge	36 (19.1%)	264 (22.3%)	χ^2^ test	*p* = 0.180

**Table 7:** Multivariable logistic regression model for predictors of 30-day readmission in acute psychiatry.

Predictor	aOR	95% CI	*p*-Value
Age (per 10-year increase)	1.05	0.97–1.13	0.210
Sex (male vs. female)	1.12	0.89–1.41	0.320
Number of previous admissions (per additional admission)	1.35	1.18–1.54	<0.001
Primary diagnosis: psychotic disorder (yes vs. no)	1.62	1.18–2.23	0.003
LOS (per 5-day increase)	0.88	0.80–0.96	0.006
Post-discharge plan documented (yes vs. no)	0.54	0.39–0.76	<0.001
LAI antipsychotic prescribed at discharge (yes vs. no)	0.79	0.58–1.08	0.140

**Table 8:** Logistic regression model performance indicators for prediction of 30-day readmission.

Performance Indicator	Value	Interpretation
Hosmer–Lemeshow goodness-of-fit	χ^2^(8) = 6.21, *p* = 0.623	Good calibration (no significant difference between observed and predicted outcomes)
Nagelkerke R^2^	0.28	Moderate explanatory power
Area under the ROC curve (AUC)	0.74	Acceptable discrimination
Sensitivity (true positive rate)	46%	Proportion of readmitted patients correctly identified by the model
Specificity (true negative rate)	82%	Proportion of non-readmitted patients correctly identified by the model
Positive predictive value (PPV)	29%	Probability that predicted readmissions are true readmissions
Negative predictive value (NPV)	91%	Probability that predicted non-readmissions are true non-readmissions

## Data Availability

Data are available on request from the corresponding author; restrictions apply due to privacy and ethical regulations.

## Abbreviations

The following abbreviations are used in this manuscript:

LOS	Length of stay
LAI	Long-acting injectable
aOR	Adjusted odds ratio
CI	Confidence interval
AUC	Area under the curve
ROC	Receiver operating characteristic
CNAS	National Health Insurance House (Romania)
ICD-10	International Classification of Diseases, 10th Revision
GDPR	General Data Protection Regulation
SD	Standard Deviation
IQR	Interquartile Range
VIF	Variance Inflation Factor
PPV	Positive predictive value
NPV	Negative predictive value
